# The Medical Work of the Local Government Board

**Published:** 1893-12-02

**Authors:** 


### The Medical Work of the Local Government Board.

### VI.—INOCULATION AND INSUSCEPTIBILITY.

Inoculation has gone through many phases since it first made its way westward and took up its abode among us. In the first place, people wilfully laid themselves open to an attack of small-pox—a slight attack—in order to avoid a more severe one. Something of the same kind occurs when parents put all their children into the same room with the one who is suffering from scarlet fever, so that they may take the disease and "get it over." The children usually take the disease, but sometimes though they "get it over," they fail to "get over it." There is a difference in the phases—a difference which means weeping and death. The primitive inoculation was sometimes as unsatisfactory in its results as this method of infection. The inoculated disease came in as severe a form as if it had been caught unintentionally. Therefore, it was readily given up in favour of vaccination.

Vaccination is the inoculation with one disease—vaccinia or cow-pox—to evade another—small-pox. Vaccinia is a slight ailment, and very few people will object to endure it if they are thereby secured from the more severe disease; and so strong are the proofs in its favour that it has been made compulsory by the law of the land. The recent form of inoculation made popular by M. Pasteur is practised on the hypothesis that a weak dose of disease will cure a severe attack of it. It is not intended to be preventive, as the old inoculation was, but absolutely curative; but it is the descendant of the old inoculation in that the disease wilfully caused and the disease intended to be cured are the same. The latest form of inoculation, on the other hand, is rather the offspring of the vaccination hypothesis—that suffering from certain diseases prevents the attack of others. The difference is, however, that whereas vaccination is merely preventive, and suffers from the disadvantage, to the popular mind, of merely keeping off a disease which might never have attacked the patient, this inoculation takes place, like Pasteur's, after infection with the other disease, the effect of which it is desired to annul. It professes also to be curative, but not on the Pasteurian or homœopathic principle that like cures like; the procedure is akin to that of using arsenic and strychnine, poisons in themselves, as remedies for certain diseases.

Experiments in this form of inoculation recorded by Dr. Klein, show, indeed, that it has not advanced very far, that there are not many ailments which we can apply the essential poison of another disease to check, but, at the same time, enough has been discovered to indicate the possibilities of this form of treatment. For example, very successful experiments have been made with the bacillus of malignant œdema and that of grouse disease. These two bacilli are much alike; they belong to the same group of organisms, and, so far as appearance goes, are hardly distinguishable from each other. On certain animals, too, their effect seems to be the same. In mice and in guinea-pigs both bacilli produce septicæmia and death, but with regard to the guinea-pigs a smaller amount of the grouse bacillus produces perceptible physiological effects. Nevertheless, when a larger quantity of both bacilli is injected, it is the œdema bacillus that is the most surely fatal in effect. In rabbits the œdema bacillus is active in its effects, while the grouse bacillus is totally inactive. It is evident, therefore, that though similar the two are not identical. Making experiments with mice, to whom both bacilli are equally fatal, Dr. Klein found that, in a large majority of cases, animals inoculated with either bacillus died; but when inoculated with equal quantities of both the grouse disease and the œdema bacilli they were not appreciably affected at the time, nor did they ail afterwards. It seems, then, that these two bacilli counteract each other.

Another experiment was dedicated to that disease which most of all arouses popular interest—tuberculosis. The first attempt was made after the manner of Koch, *i.e.*, tuberculous guinea-pigs were inoculated with a culture of tubercle. The majority died in due course, the remainder were killed; and all at the post-mortem examination showed marked tubercular degeneration, without a sign that the injection had in any way modified the course of the disease. A second series of guinea-pigs, also tuberculous, were then injected with a sterilised broth-culture of bacillus pyocyaneus. At the same time a series of guinea-pigs was inoculated with tubercle bacillus, another with a mixture of the two bacilli, while two animals were left in their previous moderately tuberculous condition for control—that is, to check the results of the other experiment. In the result all the guinea-pigs died, but those which had received the injection of bacillus pyocyaneus alone survived the others by a considerable time. At the post-mortem examination it appeared, moreover, that in the spleen were cicatrices showing where the tubercle had healed, and in the liver there were fewer of those caseous and bile-strained patches characteristic of tubercular disease than is usual. The inference is that the pyocyaneus bacillus, if it does not wholly destroy the tubercle bacillus, at least greatly retards its activity. Dr. Klein made also some experiments with calves, but found it difficult to obtain animals which were markedly tuberculous, yet in whom the disease was not so far advanced as to make the experiment use'ess. The results, therefore, though hopeful, are not decisive, and doubtless Dr. Klein will have more to say on the subject in next year's report.

A very interesting series of experiments is that made by Dr. Berry Haycraft with saliva. From these it seems clear that saliva is a sort of natural antiseptic. Dr. Haycraft observed with great care two fluids containing saliva, one of which was sterilised by boiling, the other being left in its normal condition. The fluid which had been boiled soon showed traces of putrefactive change, while the unboiled remained pure and clear for many days. It appears from this that saliva in its natural state is a ferment which prevents the action of putrefactive micro-organisms. Other experiments, made with pancreatic extract, were less decisive in result, but showed that the ferments of the body have some power, as yet unexplained, of checking the action of micro-organisms. The report concludes with the record of an interesting series of experiments by Dr. Andrewes on the power possessed by pus to kill bacteria.

